# A Deep Learning-Based Two-Branch Generative Adversarial Network for Image De-Raining

**DOI:** 10.3390/s24206724

**Published:** 2024-10-19

**Authors:** Liquan Zhao, Jie Long, Tie Zhong

**Affiliations:** Key Laboratory of Modern Power System Simulation and Control & Renewable Energy Technology, Ministry of Education, Northeast Electric Power University, Jilin 132012, China; zhaoliquan@neepu.edu.cn (L.Z.); 2202200380@neepu.edu.cn (J.L.)

**Keywords:** image de-raining, generative adversarial network, two-branch, multi-scale, residual attention

## Abstract

Raindrops can scatter and absorb light, causing images to become blurry or distorted. To improve image quality by reducing the impact of raindrops, this paper proposes a novel generative adversarial network for image de-raining. The network comprises two parts: a generative network and an adversarial network. The generative network performs image de-raining. The adversarial network determines whether the input image is rain-free or de-rained. The generative network comprises two branches: the A branch, which follows a traditional convolutional network structure, and the U branch, which utilizes a U-Net architecture. The A branch includes a multi-scale module for extracting information at different scales and a residual attention module to reduce redundant information interference. The U branch contains an encoder module designed to address the loss of details and local information caused by conventional down-sampling. To improve the performance of the generative network in image de-raining, this paper employs a relative discriminator incorporating a mean squared error loss. This discriminator better measures the differences between rainy and rain-free images while effectively preventing the occurrence of gradient vanishing. Finally, this study performs visual and quantitative comparisons of the proposed method and existing methods on three established rain image datasets. In the quantitative experiments, the proposed method outperforms existing methods regarding PSNR, SSIM, and VIF metrics. Specifically, our method achieves an average PSNR, SSIM, and VIF of approximately 5%, 3%, and 4% higher than the MFAA-GAN method, respectively. These results indicate that the de-rained images generated via the proposed method are closer to rain-free images.

## 1. Introduction

In recent years, image processing has emerged as a critical technology widely applied across multiple fields, including medicine, photography, surveillance systems, remote sensing [1], and autonomous driving. These applications heavily rely on high-quality image or video data. However, adverse weather conditions, such as rain, snow, and fog, severely obstruct and damage the images and videos captured by outdoor computer vision systems. This degradation in image quality not only affects the clarity of the videos and images but also significantly impacts subsequent tasks such as object detection [2], recognition, and scene understanding. Therefore, the adoption of advanced image processing techniques is crucial for enhancing the performance of outdoor computer vision systems. Currently, common image processing techniques include image de-raining, remote sensing image dehazing [3], low-light image enhancement [4], intelligent fault diagnosis [5], prediction of wind power [6], and image fusion. Among the various applications in image processing, the demand for single-image de-raining has become particularly urgent due to the impact of rainfall on image quality.

With the advancement of artificial intelligence, particularly deep learning, several single-image de-raining methods based on deep learning have emerged. These methods typically utilize a large number of paired images for training, consisting of both rainy and non-rainy images. Through training, an optimized network model is constructed to effectively remove raindrops and rain streaks from the images. Compared to traditional single-network deep learning approaches, Generative Adversarial Networks (GANs) demonstrate superior performance by leveraging the adversarial interplay between the generator and discriminator. Due to their unique adversarial mechanism, GAN-based methods often outperform conventional techniques in de-raining [7]. However, despite the numerous GAN-based de-raining methods proposed [8], practical applications still face several challenges. For instance, existing methods struggle to effectively restore image details and textures, often failing to adequately consider the differences between rain streaks and background information, which leads to confusion between rain and background details. Therefore, this paper proposes a two-branch generative adversarial network architecture to enhance the quality of rain-removed images and better recover the original background information.

The main contributions of this paper are summarized below:

(1) We propose a two-branch generative network for image de-raining. The network consists of two branches: the A branch and the U branch. The A branch includes our proposed multi-scale module and residual attention module. The proposed multi-scale module ensures that the network extracts information from different receptive fields. The proposed residual attention module enables the network to focus on important rain streak features while reducing interference from redundant information. The U branch contains our proposed encoder module, which improves upon the shortcomings of traditional down-sampling methods, effectively minimizing the loss of local image details.
(2) We propose an improved relative discriminator network. Based on the traditional discriminator network, we add an additional branch to calculate the error between the feature map of the discriminator network and the rain streak feature map. This error is used as supplementary supervisory information to enhance the discriminative capability of the discriminator network.
(3) We proposed an improved loss function by introducing the perceptual loss into the traditional loss function of generative adversarial networks. This enhancement allows the network to focus more on the overall content, resulting in the generation of more natural de-rained images.

## 2. Related Work

The methods for single-image de-raining can be broadly categorized into two types: traditional image de-raining methods and deep learning-based de-raining methods. Traditional image de-raining methods include image filtering-based methods, prior-based methods, and sparse coding-based methods. Among them, the image filtering-based methods remove raindrops by smoothing the image and preserving the edge information [9], but they may result in losing image details. Prior-based methods utilize prior knowledge [10], such as the dark channel, to estimate the rain intensity in the image and remove raindrops [11]. Sparse coding-based methods employ sparse coding techniques to perform de-raining on the image [12]. However, they suffer from high computational complexity and sensitivity to parameter selection and are not suitable for dealing with images containing many raindrops. Although traditional methods are relatively simple, they have limited applicability for de-raining in images and perform poorly when handling images with a significant amount of raindrops. In recent years, deep learning-based approaches have rapidly developed [13]. These methods can be divided into two categories: single image de-raining based on a conventional convolutional neural network and single image de-raining based on a Generative Adversarial Network (GAN).

For the single image de-raining based on a conventional convolutional neural network, they train network models using a large dataset of rain and rain-free images, eliminating the need for explicit rain modeling [14], which is a technique that requires the use of an explicit physical or mathematical model to simulate and generate rainfall phenomena. As a result, compared to traditional methods, they have significantly improved the effectiveness of image de-raining. However, many researchers have attempted to enhance the performance of image de-raining by designing more complex network architectures, which often leads to overfitting. To address this issue, Ren et al. proposed a de-raining architecture with recurrent convolutions [15]. This architecture achieves simple and efficient progressive cyclic de-raining by repeatedly employing a ResNet structure with recurrent layers. Jiang et al. introduced a progressive coupled network that learns joint features of the background and rain streak components [16]. The coupled representation module (CRM) gradually separates rain streaks from the background image, aiming to minimize distortion of the background content. However, the effectiveness of rain streak removal is relatively weaker in this approach.

In order to combine the advantages of deep learning and traditional methods, Wang et al. constructed the Rainband Convolutional Dictionary Network (RCDNet), which embeds the inherent prior of rain streaks, ensuring fine generalization performance even in the presence of inconsistent rain patterns between training and test data [17]. Wang et al. created a large-scale dataset of rainy and non-rainy image pairs covering a wide range of natural rain scenes and proposed a spatial attention network [18] to remove rain streaks in a local-to-global manner. RNN-based approaches are becoming a new trend, gradually removing raindrops by dividing the entire rain-removal process into multiple stages. Qin et al. proposed the multi-stage encoded feature aggregation network [19] for image de-raining, which introduced the adaptive weighted module and feature aggregation module into Convolutional Long Short-Term Memory (LSTM) to enhance the images more accurately based on masks and gradually fuse features from different layers. Zamir et al. proposed a multi-stage progressive restoration network [20] method. In the early stages, this method learns multi-scale contextual information using an encoder-decoder structure. It performs feature transformations on the original resolution images using a network structure that does not involve down-sampling in the later stages, thus preserving spatial details. The framework also introduces attention modules and cross-stage feature fusion modules to improve cross-stage progressive learning and feature propagation capabilities. Zheng et al. proposed a segmentation-aware progressive network based on contrastive learning [21] for single-image de-raining. It utilizes Progressive Dilation Units (PDUs) to significantly expand the receptive fields and learn multi-scale rain patterns and introduces novel Perceptual Contrastive Loss (PCL) and Learning Perceptual Similarity Loss (LPISL) to regulate the learning process. However, this approach relies on manually designed stage network structures and overlooks the Multi-Scale information of the images.

In single image de-raining based on GAN, the GAN consists of two components: the generator network and the discriminator network. They can indirectly improve the performance of the generator network compared to traditional single-network deep learning. Currently, GAN-based image de-raining methods are generally divided into two categories: supervised image de-raining and unsupervised image de-raining.

For GAN-based unsupervised image de-raining, Wei et al. proposed an unsupervised framework called DerainCycleGAN [22]. This framework utilizes two generators and two discriminators and applies CycleGAN’s cycle consistency and transfer learning abilities for training. They use one pair to generate de-rained images and discriminate between real rain-free and generated images. In contrast, the other pair generates raindrop streaks and discriminates between real rainy images and generated ones. Guo et al. [23] introduced an attention mechanism in the generator based on CycleGAN and a multi-scale discrimination module in the discriminator. This allows for de-raining to focus near rain lines and improve the quality of de-rained images. They also incorporated perceptual and internal perceptual loss into the loss function to make the generated images more realistic. Furthermore, Chen et al. [24] added an attention fusion module on top of the texture features in the image. This module combines channel and spatial attention to learn de-raining features effectively. Wang et al. guided the generator in producing de-raining images by generating masks. In addition, a contrast learning generator is introduced to preserve the background information of the image in order to maintain texture and semantic consistency [25]. Wedajew and Xu proposed the Recurrent Rain-Attentive Module (RRAM) rain streak extractor to detect rainfall information in images with and without rain. The Squeeze Excitation (SE) component is introduced into the generator network so that it can drive the network to learn more useful information, prevent model overfitting, and enhance the network’s generalization ability [26]. Wen Liu et al. proposed an Asymmetric Cyclic Generative and Adversarial Framework (AGCF). The framework consists of two parts: Rain-fog2Clean (R2C) and Clean2Rain-fog (C2R). An attention-based graph feature extraction network is introduced in R2C to learn potential relationships with features and backgrounds. A rain feature reorganization network is designed in C2R to ensure that richer texture details are included in the generated image [27]. Although the CycleGAN model can generate high-quality de-rained images, it still faces challenges regarding incomplete de-raining and the inability to better restore image details compared to supervised de-raining methods.

For GAN-based supervised image de-raining, Cao et al. proposed a two-stage de-raining model combining GAN and physical models [28]. In the first stage, they used a physical model to remove rain and estimate rain density preliminarily. In the second stage, they employed a conditional GAN to refine the de-rained images, thereby improving restoration quality and reducing image artifacts. Chai et al. introduced an Enhanced Attention GAN that outputs multi-scale aggregated attention maps and spatial context feature maps to a symmetric autoencoder [29]. They used the attention maps as additional supervisory information to enhance the network performance with a relative discriminator. Xue et al. proposed a multi-scale fusion self-attention GAN [30]. The network generated rain maps of different scales using a Gaussian algorithm. Then, it fused the feature maps of various scales using a designed fusion module to extract raindrop information as soon as possible. However, residual rain streaks may still exist in the de-rained images. Kolekar et al. proposed an innovative deep learning-based de-raining model [31] that employed an improved residual U-Net and a multi-scale attention-guided convolutional neural network module as the discriminator in the conditional generative adversarial network framework. The proposed method introduced custom hyperparameters and customized loss functions to facilitate effective rain streak removal from images. Yang et al. proposed a multi-scale fusion and adaptive attention generative adversarial network to remove rain streaks effectively [32]. A multi-scale feature fusion module was utilized in the generator to extract features at multiple scales. At the same time, spatial attention and channel attention were employed to capture global and local positional information. Introducing a multi-scale perceptual loss function during training reduced the artifacts caused by GAN, ensuring better visual quality. However, this network exhibited poorer de-raining performance in long-distance scenic images. Lu et al. proposed a dual U-Net generative adversarial network for image de-raining [33]. This network utilized two U-Nets with stronger learning capabilities as the generator, enabling the removal of more rain streaks accurately while preserving image details.

In general, single-image de-raining is a highly challenging problem, and methods based on GAN have provided new perspectives and directions for addressing this problem. However, image de-raining still faces challenges in effectively removing rain streaks in certain regions, resulting in residual artifacts and poor performance in detail restoration, leading to suboptimal de-raining results.

## 3. Method

To reduce the impact of rain on image quality, we propose a GAN for single image de-raining. The network is composed of a generator and a discriminator. The generator is responsible for de-raining the images, while the discriminator determines whether the input image is a de-rained image or a real rain-free image. The adversarial process between the generator and the discriminator continuously improves the generator’s de-raining capability and the discriminator’s discriminative ability. In the following sections, we will provide a detailed introduction to the generator, the discriminator, and the corresponding loss functions.

### 3.1. Proposed Generator

**Figure 1:**
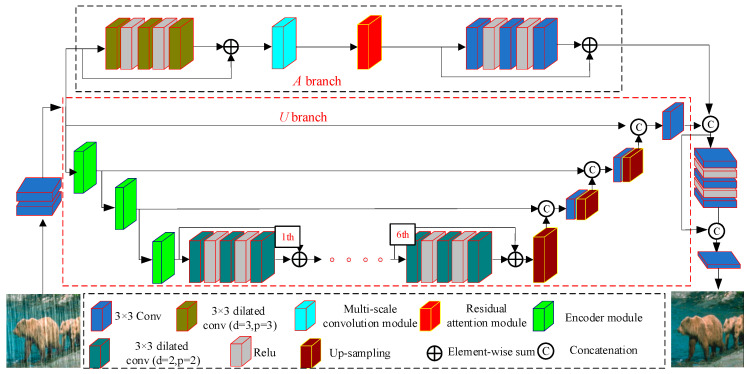
Proposed generative network.

**Figure 2:**
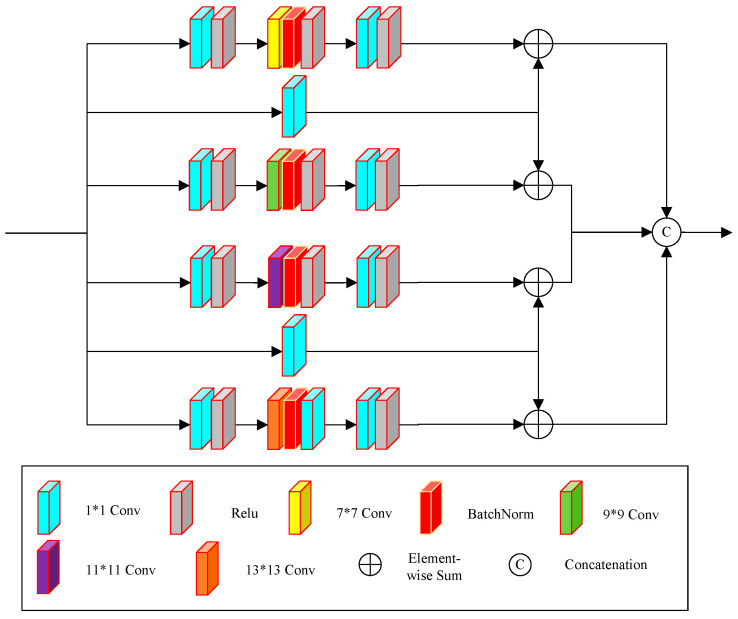
Proposed multi-scale convolution module.

**Figure 3:**
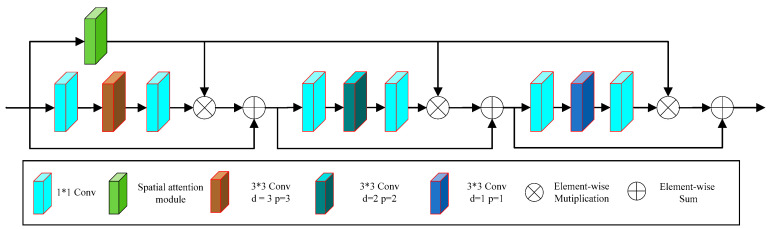
Proposed residual attention module.

**Figure 4:**
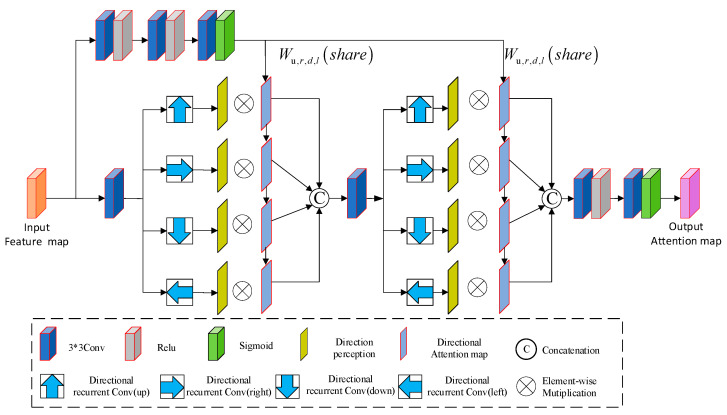
Spatial attention module.

**Figure 5:**
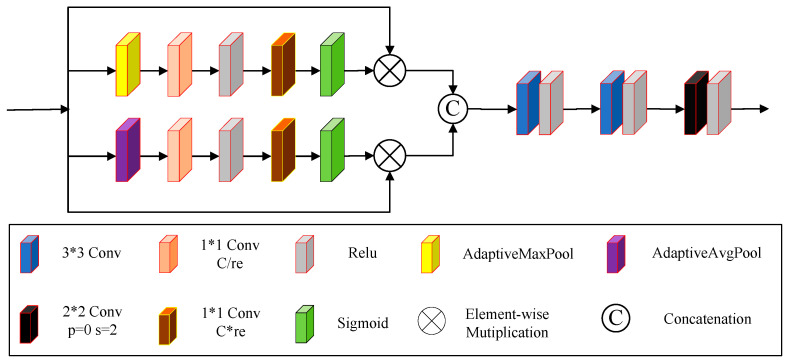
Proposed encoder module.

Figure 1 shows the structure of the proposed generator. The generator first performs initial feature extraction on the rainy image using two 3 × 3 convolutional layers, thus increasing the number of channels in the initial extracted feature map from 3 to 8. We then use the feature map as the input feature map of two parallel branches for deep feature extraction. The two parallel branches are marked as the A branch and the U branch in Figure 1. The A branch focuses on extracting relatively shallow features and includes a multi-scale convolution module (shown in Figure 2) and a residual attention module (shown in Figure 3), where the spatial attention module (shown in Figure 4) is part of the residual attention module. In contrast, the U branch focuses on extracting relatively deep features and includes an encoder module, as shown in Figure 5. After feature extraction, we fuse the outputs from the two branches through a concatenation operation. The fused features undergo further extraction and refinement through a residual network, which consists of three 3 × 3 convolutions and two ReLU activation functions. Finally, a 3 × 3 convolution is used to reconstruct the image from the extracted features, reducing the impact of raindrops on the image.

#### 3.1.1. The A Branch

The A branch show in Figure 1 primarily extracts shallow feature information, including texture and color features. It includes a multi-scale convolution module in Figure 2, a residual attention module in Figure 3, a residual module with standard convolution, and a residual module with dilated convolution. The first residual module comprises three 3 × 3 dilated convolutions, each with a dilation rate and padding of 2, and two ReLU activation functions. Compared to conventional residual networks, this structure enlarges the receptive field, facilitating the extraction of global features.

We propose the multiscale convolution module shown in Figure 2, which consists of six branches: The first branch includes a 1 × 1 convolution, a ReLU function, a 7 × 7 convolution, batch normalization, another ReLU function, a 1 × 1 convolution, and a final ReLU function. The second and fifth branches each consist of a single 1 × 1 convolution. The third branch comprises a 1 × 1 convolution, a ReLU function, a 9 × 9 convolution, batch normalization, another ReLU function, a 1 × 1 convolution, and a final ReLU function. The fourth branch includes a 1 × 1 convolution, a ReLU function, an 11 × 11 convolution, batch normalization, another ReLU function, a 1 × 1 convolution, and a final ReLU function. The sixth branch consists of a 1 × 1 convolution, a ReLU function, a 13 × 13 convolution, batch normalization, another ReLU function, a 1 × 1 convolution, and a final ReLU function. Then, we fused the output feature maps of the second branch with those of the first and third branches through element-wise addition. Similarly, the output feature maps of the fifth branch are fused with those of the fourth and sixth branches using the same method. The fused features from all branches are combined using element-wise concatenation, resulting in the output feature maps of the multi-scale convolution module.

The multi-scale module can extract information at different scales primarily because the size of the convolutional kernel directly affects the receptive field. Larger kernels have a greater receptive field, enabling the model to learn global features and enhancing its ability to perceive background and rain distribution. Conversely, smaller kernels have a smaller receptive field, allowing the network to capture local features and helping to preserve the fine details of the image. By integrating features extracted from multiple convolutional kernels of varying sizes, the network can capture information at different scales, thereby mitigating information loss due to excessive concentration in the feature space. Consequently, multi-scale convolutions can extract richer feature information.

We propose the residual attention module in the A branch to make the network focus more on rain streak features while reducing the interference of redundant features. The proposed attention module is illustrated in Figure 3, with its input being the output feature maps of the multi-scale convolution module shown in Figure 2. This attention module can be seen as composed of three serially connected hybrid modules, each consisting of three branches: a residual mapping module, skip connections, and a spatial attention module. We propose the residual mapping module using two 1 × 1 convolutions and one 3 × 3 dilated convolution to increase the receptive field and better extract local rain streak features without increasing the parameters. Additionally, to capture features at different scales, the dilation rate and padding of the dilated convolution in the residual mapping modules vary across hybrid modules (refers to the residual structure formed by the two 1 × 1 convolutions mentioned above, a 3 × 3 dilated convolution, and a spatial attention module). The dilated convolution in the first residual mapping module has a dilation rate and padding of 3, the second has a dilation rate and padding of 2, and the third has a dilation rate and padding of 1.

In this module, the input feature map first passes through the spatial attention module, generating an attention map that reflects the spatial distribution of rainfall. We then perform an element-wise multiplication between this feature map and the output feature map from the mixing module. This operation adjusts the weights of different regions in the feature map, emphasizing the importance of areas containing rain streaks while reducing the influence of background noise. Additionally, we incorporate a residual structure in this module, allowing important features to be directly passed to subsequent layers, thereby minimizing information loss. In the mixing module, we also utilize dilated convolutions with varying dilation rates to reduce the number of parameters and increase the receptive field, enabling the module to learn features at different scales. Therefore, through the learning of the residual attention module, our network can mitigate redundant information interference and enhance the significance of rain streak features.

The spatial attention module is primarily responsible for generating weights for the feature maps [17]. As shown in Figure 4, this module uses a recurrent neural network with ReLU and identity matrix initialization (IRNN) to project rain streaks in four different directions, with an auxiliary branch to capture spatial information, thus better highlighting the projected rain streaks.

The spatial attention module mainly consists of two branches. The first branch includes three layers of 3 × 3 convolutions, with the first two layers followed by ReLU activation functions and the last layer followed by a Sigmoid activation function. This branch is mainly responsible for extracting spatial context information and projecting the extracted information in four different directions. The second branch includes two rounds of four-directional IRNN architecture. The first round of IRNN performs a recursive convolution operation for four directions (up, down, left, and right) at each position on the input feature map, which can collect the horizontal and vertical neighborhood information at different positions. The second round of IRNN repeats the operation of the first round (continuing the recursive convolution of 4 different directions for each location from the feature map extracted from the first round of IRNN) and can collect the global context information and direction-aware features from the input mapping map, thus generating the global perceptual feature map. Finally, a 3 × 3 convolution and a Sigmoid activation generate the attention map. The attention map reflects the spatial distribution of rainfall and guides the subsequent rain-removal process.

#### 3.1.2. The U Branch

In conventional U-Net networks, down-sampling achieves the reduction of feature map size by using max pooling. However, max pooling only retains the most prominent features, which can result in losing image details and local information. We proposed an encoder module to mitigate this loss and replace the down-sampling and corresponding convolutional modules. Figure 5 shows the proposed encoder module. First, the encoder module uses two parallel branches to adjust the feature values of the input feature maps, assigning greater weight to important features. The first branch directs the network’s attention to salient features of the image (such as details, edges, and textures). In contrast, the second branch focuses on smooth features (such as subtle variations in brightness and color).

The first branch consists of adaptive max pooling, a 1 × 1 convolution, a ReLU function, another 1 × 1 convolution, and a Sigmoid activation function. We use adaptive max pooling to extract salient features. The second branch includes adaptive average pooling, a 1 × 1 convolution, a ReLU function, another 1 × 1 convolution, and a Sigmoid activation function. We use adaptive average pooling to extract global features. In each branch (C is the number of channels in this branch, and re is the value we set according to the number of channels), the first 1 × 1 convolution reduces the number of channels. In contrast, the second 1 × 1 convolution increases the number of channels. The Sigmoid activation function in each branch generates feature map weights. These weights are element-wise multiplied with the input feature maps to produce the output feature maps of each branch. To obtain richer features, the output feature maps of the two branches are concatenated to form a new feature map. Additionally, we use two consecutive 3 × 3 convolutions with ReLU activation functions to extract features further. Finally, a 2 × 2 convolution with a stride of 2 and a ReLU activation function is employed to down-sample the feature maps. Compared to conventional down-sampling operations, the parameters of the 2 × 2 convolution with a stride of 2 can be adaptively adjusted during training, allowing for a smoother reduction of image size while better-preserving image details and texture information, thus reducing the loss of feature information.

In the U branch, we employ three consecutive encoder modules to extract features from the image, progressively reducing the size of the feature maps with each extraction. We use six identical residual networks to extract features from the reduced-size feature maps. Each residual module within these networks consists of three 3 × 3 dilated convolutions with a dilation rate and padding of 2 and two ReLU activation functions. As the network depth increases, it can learn more complex and abstract features. To restore the feature map size, we apply three consecutive up-sampling operations. Additionally, to integrate shallow and deep features, we concatenate the up-sampled output feature maps with the corresponding output feature maps from the encoder modules. Each time features are fused, a 3 × 3 convolution is applied to adjust the number of channels and the size of the feature maps. In total, we used 3 encoder modules and 3 up-sampling operations in the U branch to obtain the final output feature map.

### 3.2. Proposed Discriminator

**Figure 6:**
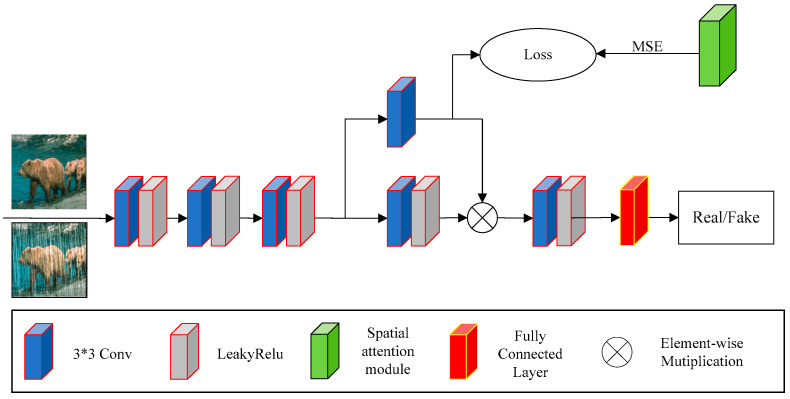
Proposed discriminator.

Conventional discriminators do not focus on specific regions of the image, which may result in failure to accurately discriminate rain streaks in de-rained images. In order to improve the discriminator’s ability to distinguish between real rain-free images and de-rained images, we propose a relative discriminator, as shown in Figure 6.

Firstly, the discriminator uses three 3 × 3 convolutions with LeakyReLU activation functions to extract features from the input image. Then, it employs a parallel convolution structure with two branches to further extract features. The first branch consists of a single 3 × 3 convolution, while the second branch comprises a 3 × 3 convolution followed by a LeakyReLU activation function. Next, the outputs from the two branches are fused using element-wise multiplication. Finally, we process the fused feature map through a sequence of a 3 × 3 convolution, a LeakyReLU activation function, and a fully connected layer to produce the classification result.

Additionally, we propose calculating the Mean Squared Error (MSE) between the output features of the first branch of the parallel convolution in the discriminator and the output features of the residual attention module in the generator. The Mean Squared Error (MSE) loss is used to measure the difference in the feature space between generated and real samples. By calculating the mean square loss of the input feature map and the attention feature map, the discriminator can adjust its parameters more finely, making the feature extraction more accurate. At the same time, it can also provide more diverse and useful gradient information for the generator. This helps the generator to better learn and tune to generate higher quality rain-free images. The residual attention module in Figure 6 corresponds to the one depicted in Figure 1. Here, the input of the residual attention module is the corresponding input rainy image, and the output is the feature attention map obtained from the rainy image. This approach leverages the difference between the feature maps of the generated and real images to measure the disparity, thereby improving the discriminator’s ability to assess the quality of the de-rained image.

### 3.3. Improved Loss Function

We propose an improved loss function to optimize the proposed GAN. This loss function can be expressed as follows:

(1) $$L=\lambda_{1}\times L_{GAN}\left(G,D\right)+\lambda_{2}\times L_{1}\left(G\right)+\lambda_{3}\times L_{per}(G)+\lambda_{4}\times L_{MSE}\left(S,M\right)$$

where $\lambda_{1}$, $\lambda_{2}$, $\lambda_{3}$, and $\lambda_{4}$ are scaling factors to adjust the importance of each loss function. The initial values of these coefficients are set based on the importance of each function. The higher the importance of the function, the larger the corresponding coefficient value. According to experimental results, the parameters are set as follows to achieve better performance: $\lambda_{1}=1$, $\lambda_{2}=0.5$, $\lambda_{3}=0.1$, $\lambda_{4}=0.1$. The $L_{GAN}(G,D)$ is the adversarial loss, ensuring that the images generated by G() are realistic enough to fool the discriminator D(). The value of adversarial loss usually ranges from a few to tens. It may be high initially, but as the training progresses, the adversarial loss will stabilize as the generator and discriminator gradually reach balance. It is expressed as follows:

(2) $$L_{GAN}\left(G,D\right)=E_{X,Y}\left[\log D(Y,X)\right]+E_{X,Y}\left[\log\left(1-D(G(X),Y)\right)\right]$$

where X represents the rainy image, Y represents the rain-free image, G() represents the generator, and the D() represents the discriminator. E[] denotes the expected values of the random variables. The value of L1 loss in the image de-raining task is usually small, generally between 0.1 and 1. The exact value depends on the resolution of the image and the complexity of the raindrops. The $L_{1}\left(G\right)$ in (1) is used to measure the accuracy of each reconstructed pixel, and its formula is expressed as follows:

(3) $$L_{1}(G)=E_{X,Y}\left[{\left\|Y-G(X)\right\|}_{1}\right]$$

The $L_{per}(G)$ in (1) is the perceptual loss, which compares high-level features of the generated and real images using a pre-trained network (e.g., VGG) to extract features. The value of the perceptual loss depends on the scale of the feature map of the pre-trained network and is usually between 1 and 10. In the image de-raining task, perceptual loss helps to preserve the structure and details of the image. This loss can be defined as:

(4) $$L_{per}(G)=E_{X,Y}\left[{\left\|\phi (Y)-\phi (G(X))\right\|}_{2}\right]$$

where ϕ(⋅) is high-level features extracted by a pre-trained VGG-19 network. The value of mean square error loss in the image de-raining task is usually small, generally between 0.01 and 0.1. A low MSE loss value suggests that the rain-removed image is very close to the target image. The $L_{MSE}(S,M)$ is our proposed loss function. It can be defined as:

(5) $$L_{MSE}(S,M)=E_{S,M}\left[{\left(S-M\right)}^{2}\right]$$

where S is the output feature map of the residual attention module in the generator. M is the output feature map of the first convolution in the parallel convolution of the discriminator.

## 4. Simulation and Discussion

In this section, we compare our method with other image de-raining techniques, specifically PCNet, SAPNet, MSFSA-GAN, and MFAA-GAN, regarding their de-raining performance. To evaluate the effectiveness of our method, we conducted experiments using the Rain100L, Rain12600, and Rain12 datasets. The Rain100L dataset contains 2000 pairs of images, with each image sized at 481 × 341 pixels. This dataset encompasses various types of raindrops and covers a wide range of natural environments, ensuring the robustness of the algorithm across different scenarios. The Rain12600 dataset consists of 14,000 pairs of images, each sized at 512 × 384 pixels. This large-scale dataset includes various rain streaks with varying intensities and types, enabling a comprehensive assessment of the algorithm’s performance under high noise conditions. Although the Rain12 dataset contains only 12 pairs of images, each sized at 481 × 341 pixels, it focuses on complex raindrop and environmental scenarios, allowing for an in-depth analysis of how each situation affects algorithm performance. In our experiments, we trained our model using 11,200 pairs of images from the Rain12600 dataset and tested it on the remaining 2800 images from the same dataset. Additionally, to test the generalization capability of our method, we evaluated it on all images from the Rain100L dataset and the entire Rain12 dataset. These three datasets encompass a variety of rainfall scenarios and intensities, providing a thorough assessment of our method’s de-raining performance.

To objectively evaluate the performance of the algorithm, we conducted a quantitative analysis by calculating the Peak Signal-to-Noise Ratio (PSNR), Structural Similarity Index Measure (SSIM), and Visual Information Fidelity (VIF) between the de-rained images and the original images. Higher values of PSNR, SSIM, and VIF indicate better de-raining performance. Additionally, our experiments are conducted on an Ubuntu 18.04 system with four NVIDIA GeForce GTX 1080 Ti GPUs, utilizing the PyTorch deep learning framework. We employed the same Adam optimizer and parameters to ensure a fair comparison among all methods, training the network for 200 epochs. During training, we set the learning rate to 1 × 10^−4^, batch size to 2, cropped the images to a size of 256 × 256, and initialized the remaining network parameters to zero. The hyperparameters are summarized in Table 1.

### 4.1. Simulation on the Rain100L Dataset

**Figure 7:**
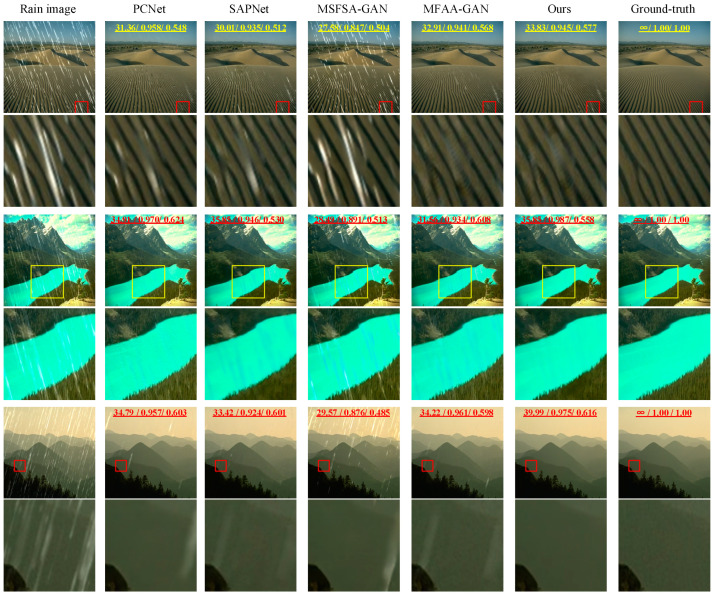
Results on Rain100L dataset.

We evaluated each method using the Rain100L dataset. We randomly selected three rainy images as test images. Synthetic rainy images, rain-free images, and de-rained images are shown in Figure 7. Among them, the numbers marked above the picture are the corresponding PSNR, SSIM, and VIF values of the picture. The first column shows rainy images, and the images from the second to the fifth column are derived from PCNet, SAPNet, MSFSA-GAN, MFAA-GAN, and our proposed method, respectively. The last column shows rain-free images. From the full-size images in the first row, it can be observed that the de-rained images obtained via MSFSA-GAN exhibit noticeable rain streaks. From the locally enlarged images in the first row, it can be seen that de-rained images obtained via SAPNet and PCNet also have some residual rain streaks. The de-rained images obtained via our proposed method, MFAA-GAN, do not exhibit noticeable residual rain streaks, but some desert-like patterns are visible in the locally enlarged images. For the second image, the de-rained image obtained via MSFSA-GAN exhibits noticeable rain streaks. In the locally enlarged images in the second row, it is evident that the de-rained image produced via the SAPNet method retains a small amount of residual rain streaks. The de-rained images generated via PCNet and MFAA-GAN do not display obvious rain streaks; however, we can observe slight residual rain streaks and artifacts in the local images. In contrast, the de-rained image obtained via our proposed method shows no residual rain streaks or artifacts. For the third image, the de-rained images obtained via the MSFSA-GAN method exhibit many residual rain streaks. Minor residual rain streaks exist in the images obtained via PCNet and MFAA-GAN methods. The locally enlarged images show that de-rained images obtained via SAPNet, PCNet, MFAA-GAN, and MSFSA-GAN methods exhibit noticeable artifacts. However, the de-rained images obtained via our proposed method do not show significant residual rain streaks and artifacts.

To objectively analyze the performance of the methods, we tested all images in the Rain100L test set. We calculated the PSNR, SSIM, and VIF for each method. Table 2 presents the results. The average PSNR values for de-rained images obtained via PCNet, SAPNet, MSFSA-GAN, MFAA-GAN, and our proposed method are 33.37, 30.75, 29.59, 34.51, and 36.37, respectively. The average SSIM values are 0.954, 0.938, 0.877, 0.956, and 0.981, respectively. The average VIF values are 0.610, 0.537, 0.533, 0.681, and 0.687, respectively. In summary, the de-rained images obtained using our method have the highest PSNR, SSIM, and VIF values. This indicates that our method has better de-raining capability compared to other methods. In addition, by comparing the PSNR, SSIM, and VIF of a single image with the average PSNR, SSIM, and VIF of all images, there is little difference between the index of a single de-rained image and the average index obtained via our method. Therefore, our method has the most stable de-raining effect.

### 4.2. Simulation on Rain12600 Dataset

**Figure 8:**
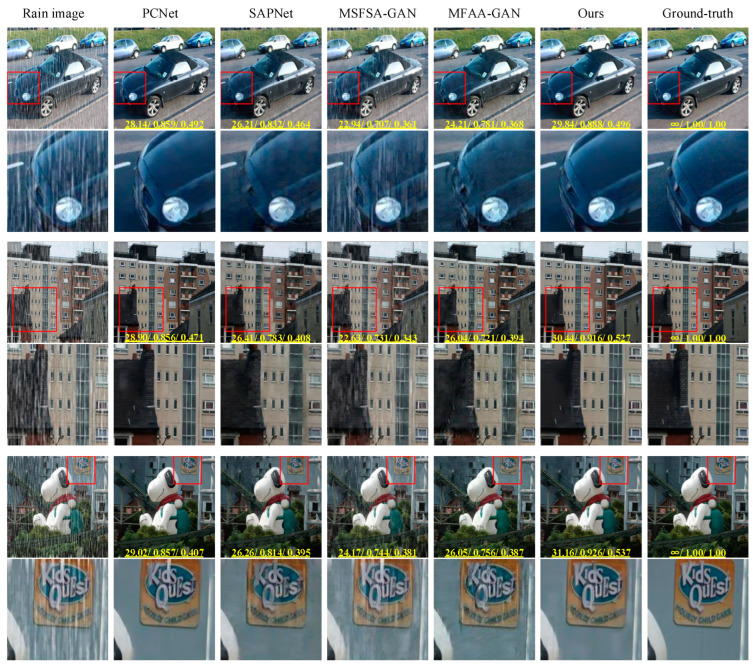
Results on Rain12600 dataset.

We evaluated each method using the Rain12600 dataset. We randomly selected three rainy images as test images. Synthetic rainy images, real rain-free images, and de-rained images are shown in Figure 8. Among them, the numbers marked below the picture are the corresponding PSNR, SSIM and VIF values of the picture. The first column shows rainy images, and the images from the second to the fifth column are derived from PCNet, SAPNet, MSFSA-GAN, MFAA-GAN, and our proposed method, respectively. The last column shows real rain-free images. From the full-size images in the first row, it can be observed that the de-rained images obtained via MSFSA-GAN exhibit noticeable rain streaks. The locally enlarged images in the second row show that the de-rained images obtained via SAPNet and MFAA-GAN have removed the rain streaks but exhibit significant artifacts. The images obtained via PCNet show minor residual rain streaks. The de-rained images obtained using our proposed method show no noticeable residual rain streaks and artifacts. In the second image, the de-rained images obtained via MSFSA-GAN exhibit noticeable rain streaks. From the locally enlarged images, it can be observed that the de-rained images obtained via SAPNet have minor residual rain streaks. The de-rained images obtained via MFAA-GAN have minor residual rain streaks but exhibit artifacts and color distortion. The de-rained images obtained using our proposed method have no residual rain streaks and artifacts and no color distortion. In the third image, the de-rained images obtained via MSFSA-GAN exhibit a large amount of residual rain streaks. The locally enlarged images show that the de-rained images obtained via MFAA-GAN have minor residual rain streaks and artifacts. The de-rained images obtained via SAPNet and PCNet exhibit noticeable artifacts and slight color distortion. The de-rained images obtained via our proposed method show no noticeable residual rain streaks, artifacts, or color distortion.

To objectively analyze the performance of the algorithms, we tested all images in the Rain12600 test set. We calculated the PSNR, SSIM, and VIF for each method. Table 3 presents the results. The average PSNR values for de-rained images obtained via PCNet, SAPNet, MSFSA-GAN, MFAA-GAN, and our proposed method are 29.97, 26.01, 24.18, 26.04, and 31.98, respectively. The average SSIM values are 0.895, 0.832, 0.757, 0.922, and 0.941, respectively. The average VIF values are 0.491, 0.414, 0.375, 0.387, and 0.524, respectively. In summary, the de-rained images obtained using our method have the highest PSNR, SSIM, and VIF values. This indicates that our method has better de-raining capability compared to other methods. In addition, by comparing the PSNR, SSIM, and VIF of a single image with the average PSNR, SSIM, and VIF of all images, we can find that the proposed method is more stable regarding the de-raining effect.

### 4.3. Simulation on Rain12 Dataset

**Figure 9:**
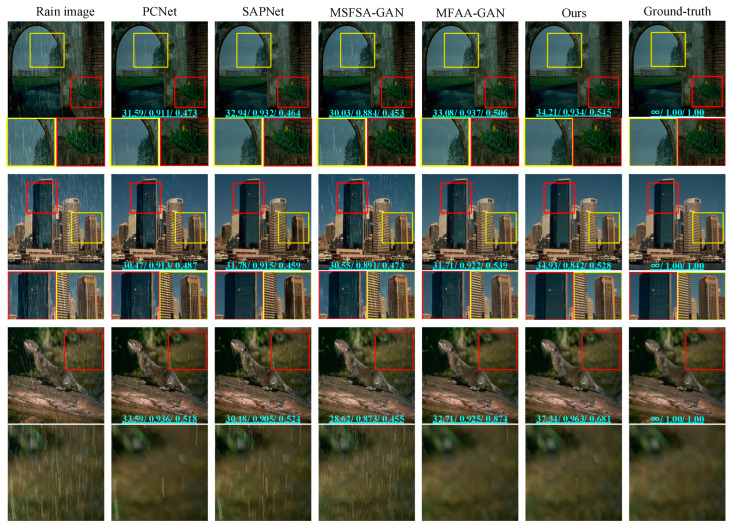
Results on Rain12 dataset.

We evaluated each method using the Rain12 dataset by randomly selecting three rainy images as test images. The synthetic rainy images, real rain-free images, and de-rained images are shown in Figure 9. Among them, the numbers marked below the picture are the corresponding PSNR, SSIM, and VIF values of the picture. The first column shows the rainy images, while the second to the fifth columns show the de-rained images obtained via PCNet, SAPNet, MSFSA-GAN, MFAA-GAN, and our proposed method, respectively. The last column displays the real rain-free images. For the first image, from the full-size images in the first row, it is evident that the de-rained images produced via MSFSA-GAN still exhibit noticeable rain streaks. In the locally enlarged images, the de-rained images generated via MFAA-GAN and PCNet also show some remaining rain streaks. However, the images derived from our proposed method and SAPNet do not show any significant residual rain streaks. In the second image, the de-rained image from MSFSA-GAN again shows noticeable rain streaks. In the locally enlarged image, the derained images from PCNet and MFAA-GAN do not show significant rain streaks but exhibit artifacts in the left image. The locally enlarged image on the right for the SAPNet method shows color distortion. The de-rained image for our proposed method does not exhibit noticeable rain streaks, artifacts, or color distortion. In the third image, the de-rained images from MSFSA-GAN and SAPNet have many remaining rain streaks. The locally enlarged images reveal that the images generated via PCNet and MFAA-GAN contain a few rain streaks. The image de-rained using the proposed method has no significant rain streaks, but some artifacts are still present compared to the real rain-free image.

To objectively analyze the performance of the algorithms, we tested all images in the Rain12 test set. We calculated the PSNR, SSIM, and VIF for each method. Table 4 presents the results. The average PSNR values for de-rained images obtained via PCNet, SAPNet, MSFSA-GAN, MFAA-GAN, and our proposed method are 32.95, 32.37, 32.46, 35.78, and 37.09, respectively. The average SSIM values are 0.931, 0.947, 0.909, 0.965, and 0.968, respectively. The average VIF values are 0.563, 0.533, 0.542, 0.614, and 0.652, respectively. In summary, the de-rained images obtained using our method have the highest PSNR, SSIM, and VIF values. This indicates that our method has better de-raining capability compared to other methods. At the same time, we compared the PSNR, SSIM, and VIF of a single image with the average PSNR, SSIM, and VIF of all the images. The comparison shows that the de-rained image index obtained via our method is the most stable.

### 4.4. Simulation on Different Rainfall Densities

**Figure 10:**
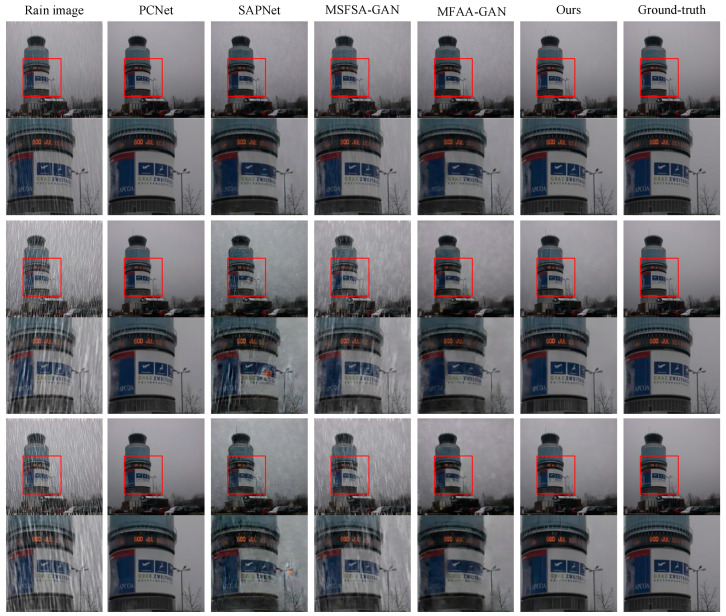
Results on different rain densities.

To comprehensively evaluate the de-raining results of images under different rain densities, we selected an image from the dataset and adjusted its rain density to low, mid, and high levels. Figure 10 presents the results of image de-raining generated using different methods at varying rain densities. A horizontal comparison reveals that our method demonstrates the best de-raining performance across all three levels of rain density. Furthermore, a vertical comparison indicates that as rain density increases, the de-raining performance of all methods, including ours, gradually declines.

To provide a quantitative assessment, we calculated the PSNR, SSIM, and VIF values for various methods, as shown in Table 5. Observing the data in the table reveals that our proposed method achieves the highest PSNR, SSIM, and VIF values, indicating that our de-raining approach is effective across different rain density levels.

### 4.5. Simulation on Real Rainfall Images

**Figure 11:**
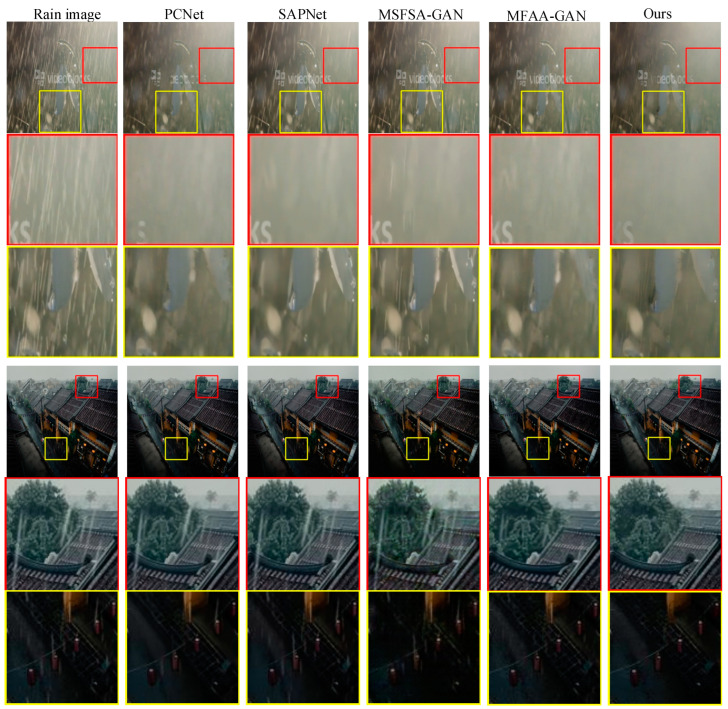
Results on real rainfall images.

To assess the generalization capability of different methods, we randomly selected two real rainy images and evaluated the de-raining performance of various approaches. Figure 11 presents the de-raining results for real rainy images. The first column displays the original rainy images, followed by the de-rained images obtained using PCNet, SAPNet, MSFSA-GAN, MFAA-GAN, and our method. In the first image, in the red region with fewer rain streaks, our method successfully removes all rain streaks, outperforming the other methods. In the yellow region of the same image, where rain streaks are more prominent, none of the methods completely eliminate all rain streaks; however, our method effectively removes most of the rain streaks and droplets, leaving only a few residual rain streaks. In the second image, the red region shows that PCNet, SAPNet, and MSFSA-GAN fail to eliminate larger rain streaks, while MFAA-GAN successfully removes these larger streaks. In contrast, our method removes most rain streaks, although a few droplets remain. The yellow region indicates that SAPNet and MSFSA-GAN can only remove a limited number of rain streaks, while PCNet and MFAA-GAN manage to eliminate most of the smaller streaks. Overall, our method demonstrates superior de-raining performance compared to others, but still leaves a few rain streaks unrecovered. Therefore, while our method achieves the best results on real images and exhibits the highest generalization capability, the complexity and diversity of real rainy images limit its ability to remove all rain streaks.

**Figure 12:**
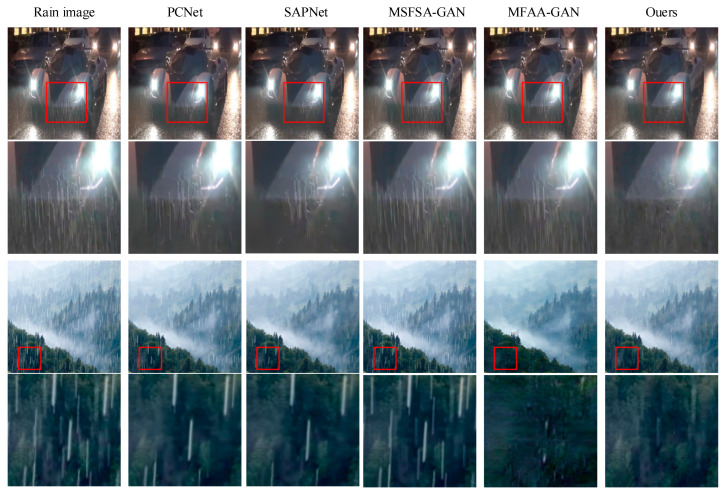
De-raining failure images.

However, the performance of our method in image de-raining is not satisfactory for certain real images, as illustrated in Figure 12. In the first image, we observe that in a nighttime scenario with rain on the road, strong light reflections combined with refracted raindrops create a white haze. While our method effectively removes most rain streaks compared to other approaches, some rain streaks and this white haze still remain in the processed image. In the second image, we note that in a scene with heavy rainfall, fog occurs, obscuring the original background with both rain streaks and haze. Although our method is able to eliminate a significant portion of the rain streaks compared to alternative methods, some rain streaks and haze persist after processing. This indicates that our approach can primarily remove rain streaks but struggles to eliminate haze in scenarios where both phenomena are present. Therefore, it is evident that our method performs poorly when handling images affected by both rain and haze. We believe this suboptimal performance in image de-raining may be attributed to the significant differences between the image scenes used in the training dataset and those that exhibit both rain and haze simultaneously.

### 4.6. Ablation Experiment

To validate the effectiveness of the proposed modules, we conducted experiments by removing each proposed module from the complete network. Specifically, we removed the multi-scale convolution, residual attention, encoder module (replaced by conventional convolution and down-sampling modules), and the feature map loss function. We used the following notation:

- No_EM: Network without the encoder module.
- No_MSCM: Network without the multi-scale convolution module.
- No_RAM: Network without the residual attention module.
- No_LS: Network without the feature map loss function.

The experiment results are shown in Table 6. As observed from the table, the PSNR, SSIM, and VIF values of the No_EM, No_MSCM, No_RAM, and No_LS methods are all lower than those of the complete network. This indicates that the proposed modules and loss functions are practical and contribute significantly to the overall network performance. No_EM: The average PSNR value is the lowest, reducing by 8% compared to the complete network. The average SSIM value is the lowest, reducing by 5%, and the VIF value is lower by 7%. No_MSCM: The average PSNR value is lower by 7%, the SSIM value is lower by 4%, and the VIF value is lower by 6%. No_RAM: The average PSNR value is lower by 9%, the SSIM value is lower by 5%, and the VIF value is lower by 10%. No_LS: The average PSNR value is lower by 3%, the SSIM value is lower by 5%, and the VIF value is lower by 7%. Among these, while keeping the two branches, the absence of the residual attention module (No_RAM) significantly impacts the network performance, resulting in the lowest PSNR and SSIM values. This suggests that the residual attention module is crucial in enhancing network performance. The residual attention module can extract essential features and reduce the influence of less critical features, effectively highlighting the rain streak information in the image, thereby improving the precision of de-raining. Therefore, this module contributes the most to the network’s performance enhancement.

## 5. Conclusions

This paper proposes a two-branch generative adversarial network for image de-raining. Firstly, we proposed a multi-scale convolution module, a residual attention module, and an encoder module, utilizing these to construct a generator network for image de-raining. Secondly, we proposed an improved discriminator network, which calculates the mean squared error between the feature maps extracted by the discriminator and the residual attention feature maps. This error is incorporated into the loss function to assist the discriminator’s decision-making process. In addition, we incorporated perceptual loss into the loss function to generate more natural de-rained images. Finally, we tested our proposed method on various rainy image datasets and compared its performance with PCNet, SAPNet, MSFSA-GAN, and MFAA-GAN methods. Our method achieves the highest PSNR, SSIM, and VIF on three synthetic rainy image datasets. These results demonstrate that our approach produces higher-quality de-rained images than other methods, generating results closest to rain-free images. Furthermore, ablation studies on the network structure and loss functions confirm the effectiveness of our method.

However, our proposed method shows a complex network structure that requires long training and testing times. In the future, we will consider simplifying the model to increase the training speed and create a more lightweight structure. In addition, our method performs poorly in processing images affected by rain and haze, with the de-raining images retaining some of the rain patterns and most of the haze. In the future, we plan to improve the network structure further and optimize the training dataset to better handle images affected by rain and haze.

## Figures and Tables

**Table 1:** Training parameters.

Training Parameters	Specifications
Patch size	256 × 256
Batch size	2
Learning rate	1 × 10^−4^
Epoch number	200
Optimizer	Adam

**Table 2:** Performance average indexes on Rain100L dataset.

Method	PSNR	SSIM	VIF
PCNet	33.37	0.954	0.610
SAPNet	30.75	0.938	0.537
MSFSA-GAN	29.59	0.877	0.533
MFAA-GAN	34.51	0.956	0.681
Our Method	36.37	0.981	0.687

**Table 3:** Performance average indexes on Rain12600 dataset.

Method	PSNR	SSIM	VIF
PCNet	29.97	0.895	0.491
SAPNet	26.01	0.832	0.414
MSFSA-GAN	24.18	0.757	0.375
MFAA-GAN	26.04	0.792	0.387
Our Method	31.98	0.922	0.524

**Table 4:** Performance average indexes on Rain12 dataset.

Method	PSNR	SSIM	VIF
PCNet	32.95	0.931	0.563
SAPNet	32.37	0.947	0.533
MSFSA-GAN	32.46	0.909	0.542
MFAA-GAN	35.78	0.965	0.614
Our Method	37.09	0.968	0.652

**Table 5:** Comparison of image de-raining performance under different rainfall densities.

		PCNet	SAPNet	MSFSA-GAN	MFAA-GAN	Our
PSNR	Low	30.97	29.15	26.04	28.96	32.72
	Mid	30.37	25.88	24.32	26.47	31.44
	High	29.01	26.26	21.62	24.21	29.17
	Average	30.17	27.09	23.99	26.55	31.11
SSIM	Low	0.932	0.886	0.842	0.875	0.954
	Mid	0.912	0.812	0.748	0.796	0.922
	High	0.866	0.795	0.703	0.743	0.877
	Average	0.903	0.831	0.764	0.804	0.918
VIF	Low	0.672	0.568	0.535	0.676	0.682
	Mid	0.503	0.453	0.452	0.486	0.586
	High	0.493	0.446	0.448	0.451	0.577
	Average	0.556	0.489	0.478	0.538	0.581

**Table 6:** Performance comparison of the method under the absence of modules.

Method	PSNR	SSIM	VIF
NO_ EM	33.47	0.935	0.637
NO_ MSCM	33.70	0.942	0.649
NO_RAM	33.01	0.932	0.620
NO_LS	35.16	0.933	0.642
Our Method	36.37	0.981	0.687

## Data Availability

Data set can apply for it at https://www.icst.pku.edu.cn/struct/Projects/joint_rain_removal.html and https://github.com/csdwren/PReNet.
